# Cardiovascular Toxicities from Systemic Breast Cancer Therapy

**DOI:** 10.3389/fonc.2014.00346

**Published:** 2014-12-04

**Authors:** Shuang Guo, Serena Wong

**Affiliations:** ^1^Rutgers Robert Wood Johnson Medical School, Rutgers, The State University of New Jersey, New Brunswick, NJ, USA; ^2^Division of Medical Oncology, Rutgers Cancer Institute of New Jersey, Rutgers Robert Wood Johnson Medical School, Rutgers, The State University of New Jersey, New Brunswick, NJ, USA

**Keywords:** cardiotoxicity, heart failure, chemotherapy, breast cancer, anthracycline, trastuzumab

## Abstract

Cardiovascular toxicity is unfortunately a potential short- or long-term sequela of breast cancer therapy. Both conventional chemotherapeutic agents such as anthracyclines and newer targeted agents such as trastuzumab can cause varying degrees of cardiac dysfunction. Type I cardiac toxicity is dose-dependent and irreversible, whereas Type II is not dose-dependent and is generally reversible with cessation of the drug. In this review, we discuss what is currently known about the cardiovascular effects of systemic breast cancer treatments, with a focus on the putative mechanisms of toxicity, the role of biomarkers, and potential methods of preventing and minimizing cardiovascular complications.

## Introduction

Breast cancer remains the most common cancer among women. It is estimated that more than 3.1 million women with a history of invasive breast cancer are alive in the United States. By 2024, that number is estimated to increase to 3.9 million (1). Advances in breast cancer treatments have fortunately led to improvements in survival. However, with more women living longer, delayed toxicities of therapy have become more significant. Cardiovascular complications from therapy can occur to varying degrees and, in severe cases, can have devastating consequences.

Cardiotoxicity from breast cancer therapy is seen most commonly after treatment with anthracyclines and trastuzumab, at times necessitating discontinuation of otherwise effective treatment. Yet despite the large number of studies that have addressed the cardiotoxic effects from breast cancer therapy, few guidelines exist for the detection, monitoring, and management of patients with treatment-related cardiotoxicity.

Cardio-oncology has emerged as a new field that focuses on preserving cardiovascular health in cancer patients. Key areas of research include understanding the mechanisms of cardiac dysfunction, developing biomarkers for early detection, and instituting appropriate therapy for both the prevention and treatment of cardiac toxicity.

## Anthracycline-Induced Cardiotoxicity

The role of anthracyclines in the treatment of breast cancer is well-established (2, 3). However, the benefit of this class of drugs is often limited by the risk of myocardial damage which, in severe cases, can progress to symptomatic congestive heart failure (CHF). Anthracycline-induced cardiotoxicity can take on a variety of forms. Acute cardiotoxicity is relatively uncommon and is usually not life threatening. It can manifest as electrocardiogram changes, arrhythmias, and transient depression of myocardial contractility. These changes generally occur during intravenous administration and normally resolve on their own with discontinuation of the drug (4–6). Chronic anthracycline-induced cardiotoxicity, on the other hand, is more common and of much greater clinical concern. This type of cardiotoxicity is classified as Type I and is dose-related, progressive and irreversible.

Chronic cardiotoxicity typically presents within 1 year of treatment but late manifestations can occur up to 10 or more years following anthracycline therapy (7). Asymptomatic diastolic dysfunction may be an early finding in patients exposed to anthracycline therapy; this may progress to heart failure with preserved left ventricular ejection fraction (LVEF) and eventually to heart failure with reduced EF. Endomyocardial biopsies performed on patients exposed to anthracyclines have demonstrated ultrastructural changes such as vacuolization, myofibrillar disorganization, and myocyte necrosis, even in the absence of overt clinical symptoms (8, 9).

The dose-dependent relationship of anthracyclines and cardiotoxicity has been well-characterized since the late 1970s. In a retrospective analysis of 4018 patients with a variety of tumors who received doxorubicin, the number of patients who developed CHF was 3% at a cumulative doxorubicin dose of 400 mg/m^2^, 7% at a cumulative dose of 550 mg/m^2^, and 18% at a dose of 700 mg/m^2^ (4). However, another analysis of doxorubicin-associated cardiotoxicity revealed an even higher incidence of CHF at 26% with a cumulative dose of 550 mg/m^2^ (10). These authors also found that CHF occurred at total cumulative doses of <300 mg/m^2^, although this was relatively infrequent. Based on these observations, it is generally recommended that the cumulative dose of doxorubicin be limited to 400–450 mg/m^2^ in adults (see Table 1).

**Table 1:** **Incidence of doxorubicin-induced CHF in the metastatic setting**.

Study	Number of patients in analysis	Malignancy	Overall incidence of CHF (%)	Incidence of CHF based on cumulative dose of doxorubicin
Von Hoff et al. (4)	4018	Variety of tumors	2.2^a^	3% at 400 mg/m^2^
				7% at 550 mg/m^2^
				18% at 700 mg/m^2^
Swain et al. (10)	630	Metastatic breast cancer and small cell lung cancer	5.1^b^	5% at 400 mg/m^2^
				16% at 500 mg/m^2^
				26% at 550 mg/m^2^
				48% at 700 mg/m^2^

*^a^Based on clinical signs and symptoms of CHF*.

*^b^Protocol definition of CHF included two or more of the following: cardiomegaly on chest X-ray; basilar rales; S3 gallop; or paroxysmal nocturnal dyspnea, orthopnea, or significant dyspnea on exertion*.

While cumulative dose remains the most significant risk factor for the development of cardiotoxicity, other risk factors include older age, radiation therapy, concomitant chemotherapy, and factors that may predispose to cardiovascular disease such as hypertension and diabetes (4, 10–13). However, there is great variability in patient susceptibility to cardiotoxicity. In the analysis by Von Hoff, five patients received over 1000 mg/m^2^ of doxorubicin and did not develop clinical cardiotoxicity, while others developed CHF at much lower cumulative doses (4). Genetic polymorphisms may account at least in part for some of the differences in susceptibility, although studies performed in the pediatric population have yielded conflicting results (14, 15).

### Mechanism of anthracycline-induced cardiotoxicity

Cardiac myocytes have limited regenerative capabilities and thus are especially susceptible to irreversible damage. The mechanism by which anthracyclines induce cardiotoxicity has been a topic of great debate and multiple hypotheses have been proposed. Until recently the most widely accepted explanation has been the oxidative stress model whereby generation of reactive oxygen species (ROS) by redox cycling and iron–anthracycline complexes leads to myocyte damage. However, this hypothesis has been called into question given the failure of antioxidants such as vitamin E, coenzyme Q10, and *N*-acetylcysteine and iron chelators such a deferasirox to confer a cardioprotective effect in the clinical setting (16–19).

More recently, investigators from MD Anderson Center proposed an alternate explanation for anthracycline-induced cardiotoxicity via the topoisomerase (Top) 2b enzyme (20). Anthracycline binding to Top2 is a well-established mechanism of cellular damage and antitumor activity. Top2a is overexpressed in cancer cells but is absent in normal cells, whereas Top2b is expressed only in normal cells, including cardiac myocytes. Using a mouse knockout model, the researchers demonstrated that cardiomyocyte-specific deletion of Top2b conferred protection against doxorubicin-induced DNA damage, mitochondrial dysfunction and generation of ROS. In addition, mice lacking the Top2b gene in cardiac muscle did not develop progressive heart failure, whereas those with intact Top2 showed a decrease in EF after doxorubicin exposure. Based on these findings, the investigators are now evaluating the utility of a Top2 blood test in predicting sensitivity to doxorubicin-induced cardiac toxicity. If the Top2 hypothesis is confirmed, one potential alternative to avoid doxorubicin-induced cardiotoxicity could be to develop Top2a-specific anthracyclines that would have maximum antitumor activity without causing cardiotoxicity.

### Cardioprotectants

Several strategies have been used in an attempt to minimize anthracycline-induced cardiotoxicity. Dexrazoxane is a cardioprotective agent that is currently indicated for reducing the incidence and severity of cardiomyopathy associated with doxorubicin administration in patients with metastatic breast cancer, who have received a cumulative doxorubicin dose of 300 mg/m^2^ and who will continue to receive doxorubicin therapy to maintain tumor control. Multiple studies have demonstrated a cardioprotective effect when this agent is used during anthracycline treatment for advanced breast cancer (21–25). In all of these studies, patients who received dexrazoxane had a decreased incidence of CHF compared to those who did not receive the drug. Yet despite these consistent positive findings, the use of dexrazoxane has not been widely adopted. This is due in part to the suggestion from a single study that dexrazoxane may lower response rates, although there was no impact on time to progression (TTP) or overall survival (OS) seen in that trial (23). This finding has not been seen in other studies, and a meta-analysis of five randomized trials (*n* = 818) showed that there was no difference in response rates between control patients and patients receiving dexrazoxane (26).

The mechanism by which dexrazoxane is thought to confer cardioprotection has not been fully elucidated. By binding to the anthracycline–iron complex and removing iron, dexrazoxane may prevent free radical-induced lipid peroxidation of mitochondrial membranes and endoplasmic reticulum (27, 28). However, this theory of iron chelation does not fully explain the cardioprotective mechanism of the drug given the observation that deferasirox, an efficient iron-chelating agent that has been shown to enter myocytes and displace iron from the anthracycline-iron complex, is unable to protect myocytes from doxorubicin damage (19, 28). An alternate hypothesis involves the ability of dexrazoxane to prevent anthracycline binding to Top2. As discussed above, when doxorubicin binds to Top2b in cardiac myocytes, it leads to DNA damage, activation of the apoptotic pathway, and induction of transcriptome change that leads to generation of ROS. Dexrazoxane binds to both Top2a and Top2b, thereby preventing anthracycline binding of Top2 and consequent doxorubicin-induced cell death (29). This hypothesis is supported by the observation that ICRF 161, an analog of dexrazoxane with iron-chelating properties but lacking activity against Top2, is unable to confer cardioprotection in rat models (30).

### Reducing the risk of toxicity

Other strategies to reduce cardiotoxicity have also been evaluated. Continuous infusion of doxorubicin is associated with less cardiac toxicity than bolus infusion (31, 32). However, this schedule is less convenient and hence is not a widely used strategy.

4′-Epidoxorubicin, or epirubicin, is an analog of doxorubicin with equivalent efficacy and less cardiotoxicity on a milligram to milligram comparison (33–36). In a prospective randomized comparison of doxorubicin and epirubicin in patients with breast cancer, epirubicin was associated with a longer median duration of response at 11.9 months compared to 7.1 months with doxorubicin. The cumulative doses at which CHF occurred was 1134 mg/m^2^ with epirubicin compared to 492 mg/m^2^ with doxorubicin (15). In a meta-analysis of 13 studies comparing doxorubicin with epirubicin, the majority of which included women with advanced or metastatic breast cancer, epirubicin was associated with a significantly decreased risk of clinical cardiotoxicity, subclinical cardiotoxicity, and any cardiac event compared to doxorubicin (32). Epirubicin-induced cardiotoxicity is also dose-dependent (37), and the United States Food and Drug Administration (FDA) recommends a maximum cumulative dose of 900 mg/m^2^. However, in an analysis of 1097 patients, the safe maximum cumulative dosage was found to be lower when risk factors such as age, radiation, and underlying cardiac risk factors were taken into consideration (38).

Altering the pharmacokinetic and pharmacodynamic profile of doxorubicin via liposome encapsulation has been an extremely effective strategy to minimize cardiotoxicity and maximize efficacy (39). One formulation of liposomal doxorubicin (Myocet) has polyethylene glycol embedded in the lipid layers, allowing the drug to stay in the liposome until it reaches the target tumor site (40). It is thought that the small size of the liposomes enables the drug to penetrate the compromised vasculature of tumors, allowing preferential delivery of the drug to the target tumor. Pegylation protects the liposome from uptake by the reticular-endothelial system, thereby increasing blood circulation time. Pegylated liposomal doxorubicin (PLD [Doxil/Caelyx]) has a half-life of 73.9 h; this prolonged circulation time enables greater uptake of the drug by tumor. A phase III trial was conducted in 509 women with metastatic breast cancer to compare the efficacy and safety of PLD and conventional doxorubicin in the first-line setting. Response rates, progression-free survival (PFS), and OS were similar in both arms; however, overall risk of cardiotoxicity was significantly higher with conventional doxorubicin than with PLD (*P* < 0.001, HR = 3.16) (41). In a meta-analysis of four studies comparing liposomal doxorubicin with conventional doxorubicin for the treatment of breast cancer or multiple myeloma, liposomal doxorubicin appeared to have similar efficacy to doxorubicin but was associated with significantly less risk of clinical or subclinical cardiotoxicity or any cardiotoxic event (32).

## Trastuzumab-Induced Cardiotoxicity

Approximately 20–25% of breast cancers overexpress the human epidermal growth factor receptor-2 (HER2 or ErbB2) (42, 43). Such overexpression has been associated with more aggressive tumor biology, altered responsiveness to therapy, and poor clinical outcome including shortened survival (44). The development of trastuzumab, a humanized monoclonal antibody that targets the HER2 receptor, has been a major advance in the treatment of HER2-positive breast cancer.

The pivotal trial that led to the FDA approval of trastuzumab in 1998 enrolled 469 patients with previously untreated HER2-positive metastatic breast cancer who were randomized to receive chemotherapy alone (consisting of an anthracycline plus cyclophosphamide in anthracycline-naïve patients or paclitaxel in patients previously treated with an anthracycline) or chemotherapy plus trastuzumab (45). The combination of chemotherapy plus trastuzumab resulted in an improved response rate (50 versus 32%; *P* < 0.001), TTP (median 7.4 versus 4.6 months; *P* < 0.001), and OS (median 25.1 versus 20.3 months; *P* = 0.046) when compared with chemotherapy alone.

An unanticipated serious adverse event that emerged from the study was cardiac dysfunction. The rates of cardiac toxicity in patients randomized to doxorubicin and cyclophosphamide (AC) with trastuzumab versus AC alone were 27 and 8%, respectively, and the rates of cardiac dysfunction in patients who received paclitaxel and trastuzumab versus paclitaxel alone were 13 and 1%, respectively. The incidence of New York Heart Association (NYHA) class III or IV heart failure was highest among patients receiving AC plus trastuzumab: 16%, compared with 3% for patients receiving AC alone, 2% for paclitaxel plus trastuzumab, and 1% for paclitaxel alone. Because the combination of trastuzumab plus doxorubicin resulted in unacceptably high rates of cardiac toxicity, these agents are generally not administered concurrently unless within the context of a clinical trial.

As a result of the above findings, the major adjuvant trials allowed only sequential administration of anthracyclines and trastuzumab (46–54). In addition, patients with abnormal cardiac function were excluded, the cumulative doxorubicin dose was limited to 300 mg/m^2^, and strict cardiac monitoring was mandated. The incidence of severe cardiotoxicity (NYHA class III or IV) in the adjuvant trials was modest: 0–4.1% in trastuzumab-treated patients versus 0–1.3% in the non-trastuzumab population (see Table 2). A meta-analysis of the five major adjuvant trials reported a 2.5-fold higher risk of cardiotoxicity with trastuzumab (54). Not surprisingly, a higher incidence of cardiotoxicity has been noted in patients also treated with anthracyclines and in patients who received a longer duration of trastuzumab in the adjuvant setting (55, 56).

**Table 2:** **Incidence of trastuzumab-associated cardiac events in adjuvant breast cancer trials**.

Trial	Number of patients in analysis	Treatment arm	Incidence of cardiac events (%)	Definition of cardiac event
NSABP B-31 (46, 51)	814	AC → P	0.8	NYHA class III/IV CHF or possible/probable cardiac death
	850	AC → PH	4.1	
NCCTG N9831 (52)	664	AC → P	0.3	Symptomatic CHF or probably/definite cardiac death
	710	AC → P → H	2.8	
	570	AC → PH	3.3	
HERA (53)	1744	Chemotherapy	0.1	NYHA class III/IV CHF with decrease in LVEF ≥10% from baseline to LVEF <50%; or cardiac death
	1682	Chemotherapy + 1 year of Trastuzumab	0.8	
	1673	Chemotherapy + 2 years of Trastuzumab	1.0	
BCIRG 006 (48)	1073	AC → T	0.7	NYHA class III/IV CHF
	1074	AC → TH	2	
	1075	TCH	0.4	
FinHer (95)	116	Chemotherapy	1.7	Symptomatic heart failure
	115	Chemotherapy + Trastuzumab	0.9	

*AC, doxorubicin + cyclophosphamide; P, paclitaxel; H, trastuzumab; T, docetaxel; C, carboplatin*.

Risk factors for the development of trastuzumab-related cardiotoxicity include prior or concurrent anthracycline use, age >50 years, pre-existing cardiac dysfunction, use of anti-hypertensive medication, and higher body mass index (56–59). The risk appears to be highest when trastuzumab is administered concurrently with anthracyclines, mostly when the cumulative dose of doxorubicin exceeds 300 mg/m^2^ (60). Cumulative doses up to 180 mg/m^2^ may in fact be safe to administer concurrently with trastuzumab (61), although there are no data demonstrating that such an approach would be more effective than sequential administration.

### Mechanism of trastuzumab-induced cardiotoxicity

Unlike anthracycline-induced cardiotoxicity, trastuzumab-related cardiotoxicity is classified as Type II and is clinically and mechanistically distinct (62). Type II cardiotoxicity is not dose-dependent, is highly reversible, and is not associated with ultrastructural changes. This was demonstrated in a study of 38 patients with suspected trastuzumab-related cardiotoxicity, all of whom had received prior doxorubicin (63). Mean LVEF prior to initiation of trastuzumab was 61%, decreased to 43% after trastuzumab, and increased to 56% after withdrawal of trastuzumab. Increases of LVEF were seen in 37 out of the 38 patients and mean time to recovery was 1.5 months. Six of the patients recovered without medical treatment. Nine patients underwent endomyocardial biopsy and the ultrastructural changes typical of anthracycline damage were not seen. The absence of such changes on biopsy demonstrates that the mechanism by which trastuzumab induces cardiac dysfunction is different from anthracyclines and is likely due to myocardial stunning or hibernation.

The pathogenesis of trastuzumab cardiotoxicity is not completely understood. The HER2 signaling pathway is essential for cardiac development and function (64), and mouse models lacking HER2 exhibit multiple characteristics of dilated cardiomyopathy, including chamber dilation, wall thinning, and decreased contractility (65). HER2 signaling is upregulated in animal models when the myocardium is under stress (66) Binding of trastuzumab to HER2 is thought to disrupt HER2-HER4 heterodimerization, thus disabling the protective mechanisms in the cardiac myocyte that are essential during exposure to adverse conditions or cardiac toxins (67). This hypothesis is consistent with the observation that increased cardiac toxicity is seen when trastuzumab is used in association with anthracyclines. Indeed, HER2-deficient cardiac myocytes are more susceptible to anthracycline-induced damage (65, 68). Activation of HER2 signaling by neuregulin-1 has been shown to improve cardiac myocyte function and survival, and upregulation of this pathway in the heart may be a potential therapeutic approach (69).

### Clinical use of trastuzumab

The risk of cardiac toxicity with trastuzumab needs to be considered within the clinical context. In the metastatic setting, the benefits conferred by the addition of trastuzumab often outweigh the potential cardiac risks. On the other hand, in the early-stage setting, a significant number of patients will be cured with local therapy alone and may not in fact require adjuvant systemic therapy. However, since it is not currently possible to identify these patients, adjuvant therapy is offered to many who may not in fact derive benefit. One must therefore be especially judicious when selecting an adjuvant regimen, which may expose a patient to unnecessary toxicities, including CHF.

Because of the increased risks of cardiac toxicity seen with both the anthracyclines and trastuzumab, there has been much interest in the development of non-anthracycline-containing trastuzumab regimens. Of the pivotal adjuvant trastuzumab trials, Breast Cancer International Research Group (BCIRG) 006 was the only one to include a non-anthracycline-containing arm (48). In this study, 3222 patients with HER2+ breast cancer were randomized to receive AC followed by docetaxel (AC-T), AC-T plus trastuzumab initiated concurrently with docetaxel (AC-TH), or docetaxel + carboplatin + trastuzumab (TCH). No significant differences in DFS and OS were found between the two trastuzumab regimens. However, there were significant differences in the incidence of CHF: 0.4% in the AC-T arm, 2.0% in the AC-TH arm, and 0.7% in the TCH arm. In addition, AC-TH was associated with a significantly increased risk of persistent decline in EF at 4 years compared to TCH.

A recent retrospective population-based cohort study evaluated the long-term risk of heart failure (in this case defined as hospitalization or two ambulatory visits within 12 months) associated with adjuvant trastuzumab and chemotherapy (70). Women included in this study were diagnosed with early-stage breast cancer between 2003 and 2009. Those with metastatic breast cancer or a pre-existing diagnosis of heart failure were excluded. 19,074 women treated with chemotherapy were identified, of whom 18% also received adjuvant trastuzumab. After a median follow-up of 5.9 years, investigators found that adjuvant trastuzumab was associated with an increased risk of heart failure (5.3 versus 2.6%, *P* < 0.0001). However, the increased risk was seen only within the first 1.5 years of treatment (HR = 5.77, 95% CI 4.38–7.62, *p* = 0.0004); thereafter, there was no difference in the risk of developing CHF between women treated with trastuzumab plus chemotherapy and those treated with chemotherapy alone (HR = 0.87, 95% CI 0.57–1.33, *p* = 0.53). These data are reassuring in that the risk of trastuzumab-associated cardiotoxicity appears to be limited to the period of active treatment.

No evidence-based guidelines exist for cardiac monitoring while on trastuzumab therapy. The FDA-approved manufacturer’s package insert recommends that a baseline assessment of cardiac function be performed prior to the initiation of therapy and LVEF measurements should be repeated every 3 months during and upon completion of therapy (71). If trastuzumab is withheld for significant cardiac dysfunction, LVEF measurement should be repeated at 4-week intervals. In the adjuvant setting it is recommended that LVEF be assessed every 6 months for at least 2 years following completion of therapy. In the metastatic setting, however, symptom-triggered evaluation of LVEF may be more appropriate given the risk-benefit ratio.

Trastuzumab should be held for 4 weeks if LVEF declines ≥16% from baseline or if there is a ≥10% decrease in LVEF from baseline to below the lower limit of normal. Treatment may be restarted if LVEF returns to normal within 4–8 weeks. Trastuzumab should be discontinued in the presence of symptomatic heart failure.

## Cardiovascular Risk with other HER2-Directed Therapies

Lapatinib is a reversible small tyrosine kinase inhibitor that targets both the epidermal growth factor receptor (EGFR or HER1) and HER2. It is indicated for use in combination with capecitabine for the treatment of metastatic HER2+ breast cancer after progression on an anthracycline, taxane and trastuzumab. In the phase III trial that led to its approval, asymptomatic declines in EF occurred in 4 of 164 (2.4%) of patients receiving lapatinib plus capecitabine versus 1 of 152 (0.7%) of patients receiving capecitabine alone. All four patients who experienced a cardiac event on lapatinib recovered and there were no symptomatic events (72) (see Table 3). It is important to note that the patients in this trial were highly selected as all had previously received trastuzumab and cardiac dysfunction was an exclusion criterion.

**Table 3:** **Incidence of cardiac events with other HER2-directed therapies**.

Trial	Number of patients in analysis	HER2-directed therapy	Incidence of cardiac events (%)	Definition of cardiac event
Geyer et al. (72)	161	Capecitabine	0.7	Symptomatic decline in LVEF or decrease ≥20% from baseline to below institution’s lower limit of normal
	163	Lapatinib plus Capecitabine	2.4	
ALTTO (74)	2097	Trastuzumab alone	0.86	NYHA Class III/IV CHF or cardiac death
	2091	Trastuzumab followed by Lapatinib	0.25	
	2093	Trastuzumab concurrent with Lapatinib	0.97	
CLEOPATRA (76)	397	Trastuzumab + docetaxel plus placebo	6.6	LVEF decline to <50% with decrease ≥10% from baseline
	407	Trastuzumab + docetaxel plus Pertuzumab	3.8	
EMILIA (77)	445	Lapatinib + capecitabine	1.6	LVEF decline to <50% with decrease ≥15% from baseline
	481	T-DM1	1.7	

In a retrospective analysis of 3689 patients with various solid tumor types treated with lapatinib, only 1.6% of patients developed a cardiac event (73). Similar rates of cardiotoxicity were noted in patients who were pretreated with anthracyclines or trastuzumab compared to those who were not pretreated. Most of the cardiac events were usually asymptomatic and reversible, indicating a Type II cardiac toxicity. More recently, data were presented from the Adjuvant Lapatinib and/or Trastuzumab Treatment Optimization (ALTTO) trial in which patients with early-stage HER2+ breast cancer were randomized to receive adjuvant chemotherapy with trastuzumab, chemotherapy with concurrent trastuzumab and lapatinib, or chemotherapy with sequential trastuzumab and lapatinib (74). The incidence of NYHA Class III/IV heart failure was <1% in all arms, with 97% of all patients having received an anthracycline.

Pertuzumab is a humanized monoclonal antibody that binds to HER at its dimerization subdomain. When combined with trastuzumab and docetaxel in the first-line setting for metastatic HER2+ breast cancer, there was an improvement in PFS and OS compared to trastuzumab and docetaxel (75). Interestingly, in a cardiac analysis of the trial, left ventricular dysfunction was numerically higher in the control arm (8.3 versus 4.4% for all grades). Declines in EF of at least 10% from baseline to <50% occurred in 6.6 versus 3.8% of patients in the placebo and pertuzumab arms, respectively (76). Thus the combination of both antibodies did not increase the risk of cardiac adverse events.

Ado-trastuzumab emtansine (T-DM1) is an antibody-drug conjugate which combines trastuzumab with the cytoxic agent emtansine. The EMILIA trial randomized 991 patients with advanced breast cancer previously treated with trastuzumab and a taxane to TDM-1 versus lapatinib plus capecitabine. Results demonstrated an improvement in PFS and OS with T-DM1 compared to lapatinib plus capecitabine. Cardiotoxicity was 1.7% with T-DM1 versus 1.6% in the other arm (77).

## Cardiovascular Risk with Bevacizumab

In recent years, there has been considerable interest in the use of antiangiogenic agents for the treatment of various cancers. Bevacizumab, a monoclonal antibody targeting vascular endothelial growth factor (VEGF), was initially granted accelerated approval in 2008 for metastatic breast cancer based on impressive results in the first-line setting when combined with paclitaxel (78). However, while subsequent trials in the first-line metastatic setting confirmed a benefit in PFS, the magnitude of benefit was small, and no OS benefit was shown in any of the studies (79, 80). Three subsequent large adjuvant studies also failed to demonstrate a benefit in DFS or OS and consequently the FDA withdrew approval for bevacizumab in 2011. Nevertheless, as this agent is still used widely in other types of solid tumors, familiarly with its potential cardiac toxicities is important.

A meta-analysis of 5 randomized trials in metastatic breast cancer showed a statistically significant increased risk in grade 3 or higher hypertension (9.71 versus 0.64%, OR = 12.76) and left ventricular dysfunction (1.73 versus 0.78%, OR = 2.25) in patients treated with bevacizumab compared to those who did not receive the drug (81). Similar increased risks were also seen in other studies, including the three adjuvant trials (82–84). The mechanism of bevacizumab-induced hypertension is not known but may be related to nitric oxide: VEGF is thought to increase production of nitric oxide, resulting in vasodilation (85). Inhibition of nitric oxide by bevacizumab therefore leads to vasoconstriction and consequent hypertension. The mechanism of CHF is also unclear, but the development of secondary hypertension itself could be a contributing factor.

## Biomarkers

Current methods for detecting cardiotoxicity rely on evaluation of LVEF by either echocardiography or Multiple Gated Acquisition (MUGA) scan. However, by the time a decrease in EF is detected there has already been considerable myocardial damage. There is therefore a need to develop biomarkers that enable the early identification of cardiac deterioration. Such a strategy would allow for the implementation of preventive measures prior to the development of functional impairment.

Cardiac troponins I (cTnI) and T (cTnT) are sensitive and specific biomarkers of myocardial damage, whereas B-type natriuretic peptide (BNP) is a marker of volume overload. These markers have been studied as potential indicators of treatment-related cardiotoxicity.

In a study of 204 patients with a variety of malignancies receiving high-dose chemotherapy, investigators measured levels of cTnI in the plasma after every cycle. Patients were divided into troponin positive (cTnI+) and troponin negative (cTnI−) groups. In the cTnI− group, LVEF decreased transiently after chemotherapy, with a nadir after 3 months, but later recovered. On the other hand, patients in the cTnI+ group sustained greater reductions in LVEF, and the declines persisted even at the end of 7 months’ follow-up (86). The same group of investigators also evaluated the significance of persistent elevations in cTnI+ and found that patients treated with high-dose chemotherapy who had persistent increases in cTn1 had a higher incidence of cardiac toxicity (85%) compared to patients with transient increases (37%) or who had no elevation in cTn1 (1%) (87). Similarly, persistent proBNP elevation following chemotherapy has been associated with significantly lower LVEF (88). Given the heterogeneity of the study populations, however, definitive conclusions cannot be drawn at this time and more research is needed to determine whether these findings can be replicated.

## Treatment and Prevention of LV Dysfunction

Beta-blockers and angiotensin converting enzyme (ACE) inhibitors are standard treatments that have been shown to improve outcomes in patients with heart failure from a variety of etiologies. Treatment of cardiac dysfunction resulting from antineoplastic therapy follows the general cardiology guidelines for heart failure, although this practice seems to be based primarily on extrapolation rather than on evidence specifically addressing heart failure in the cancer population.

In a study of 201 consecutive patients with anthracycline-induced CHF with LVEF ≤45%, enalapril and, when tolerated, carvedilol were initiated as soon as LVEF impairment was detected (89). The investigators found that time elapsed from the end of chemotherapy to the start of heart failure therapy was a crucial variable for recovery of cardiac function. Among patients treated within 2 months after the end of chemotherapy, 64% had a complete recovery of LVEF. After 2 months, however, the percentage of patients decreased, with no complete recovery seen after 6 months.

There has been significant interest evaluating the role of beta-blockers and ACE inhibitors as prophylactic agents in patients who are at risk of developing treatment-related cardiotoxicity. In a study that enrolled 50 patients receiving anthracycline therapy for a variety of cancers, patients were randomized to receive prophylactic carvedilol or placebo (90). Baseline LVEF was similar in both groups prior to initiation of chemotherapy. At 6 months of follow-up, one patient in the carvedilol group had EF < 50% compared to five patients in the control group. The mean EF in the carvedilol group remained unchanged at the end of follow-up (70.5 versus 69.7%) but was significantly lower in the control group (68.9 versus 52.3%). However, there were some discrepancies in the reported anthracycline doses used in the study and caution should therefore be exercised when interpreting these data.

In a trial by Cardinale et al., 114 patients receiving high-dose chemotherapy and deemed at high risk for cardiotoxicity based on troponin I elevation were randomized to receive the ACE inhibitor enalapril or not (91). Enalapril was started 1 month after chemotherapy and continued for 1 year. The primary endpoint was an absolute decrease of >10% in LVEF, with a decline below the lower limit of normal. Forty-three percent of patients in the control arm met the primary endpoint, compared to none in the treatment group (*P* < 0.001). These results suggest that in patients with evidence of early cardiac damage as measured by elevated troponin I values, early institution of ACE inhibitor therapy may prevent the progression of cardiac toxicity. The prevention of cardiac dysfunction during adjuvant breast cancer therapy (PRADA) trial is an ongoing randomized, placebo-controlled study evaluating the role of beta-blockers and ACE inhibitors in preventing cardiac dysfunction during adjuvant breast cancer therapy (92).

## Lack of Clinical Guidelines

Unfortunately, the American College of Cardiology and the American Heart Association have not provided any guidelines for the detection, prevention, monitoring, or treatment of cardiotoxicity from antineoplastic therapy. All patients receiving potentially cardiotoxic anticancer drugs are considered candidates for Stage A heart failure, meaning they are at increased risk of developing cardiac dysfunction (93). The European Society of Medical Oncology has published a comprehensive set of clinical practice guidelines for the management of patients with cardiotoxicity from chemotherapy, targeted agents, and radiation (94). A similar consensus statement from the major American cardiology societies would be extremely helpful to practicing clinicians to ensure maximal anticancer benefits from therapy with minimal cardiac complications.

## Conclusion

While much progress has been made in the treatment of breast cancer, cardiac complications resulting from therapy remain a significant concern. Both anthracyclines and novel targeted agents can inflict cardiac damage, although the mechanisms by which they do so and their clinical manifestations appear to be distinct. The challenge for the future will be to develop methods for early detection of cardiac dysfunction, identify strategies for prevention and treatment of cardiotoxicity, and establish clinical guidelines for practicing physicians. Many questions remain unanswered, and ongoing research and collaboration between oncologists and cardiologists are needed to ensure optimal efficacy and safety of current and future anticancer agents.

## Conflict of Interest Statement

The authors declare that the research was conducted in the absence of any commercial or financial relationships that could be construed as a potential conflict of interest. The Guest Associate Editor Sharad Goyal declares that, despite being affiliated to the same institution as author Shuang Guo & Serena Wong, the review process was handled objectively and no conflict of interest exists.
